# Liminality and the Limits of Law in Health Research Regulation: What are we Missing in the Spaces in-Between?

**DOI:** 10.1093/medlaw/fww029

**Published:** 2016-12-08

**Authors:** Graeme Laurie

**Affiliations:** 1 School of Law, University of Edinburgh, Edinburgh, Scotland

**Keywords:** Health research, Liminality, Regulation, Confidentiality, Consent

## Abstract

This article fundamentally challenges the way in which law currently regulates human health research. It invokes the anthropological concept of *liminality*—the quality of in-between-ness—to suggest deeper ways of understanding ongoing challenges in delivering acceptable and effective regulation of research involving human participants. In stark contrast to the structural regulatory spaces constructed by law, the metaphor of the *liminal space* is explored to explain what is lost through our failure to see health research regulation as an inherently human experiential process, involving potentially profound transformative events for participants and researchers alike. The implications for the future of health research regulation are then examined. In particular, the analysis calls into question key features of the current regulatory paradigm, and demands that we reconsider our own demands of law in this context. The argument is made that health research is a liminal process and that we fail to treat it as such. This requires a rethink of corollary regulation also in processual terms. Ultimately, the charge is to undertake a radical reimagining of regulatory space to accommodate and promote *liminal regulatory spaces*.

## I. INTRODUCTION: UNDERSTANDING REGULATORY SPACE IN HEALTH RESEARCH

The metaphor of the ‘regulatory space’^1^ is now commonly invoked to describe the metaphysical environment occupied by institutional actors and bounded by law. It delineates a sphere of influence and control over many human endeavours in a diverse range of contexts. This is equally true in the realm of health research regulation, where we have witnessed a burgeoning of legal and quasi-legal interventions in recent decades, nationally and internationally, and sadly too often driven by public scandals or moral panics. To take the British and European context as an example, the UK was the first country anywhere to regulate the creation, storage, and use of human embryos through the introduction of the Human Fertilisation and Embryology (HFE) Act 1990^2^ in the wake of the birth of the first so-called test-tube baby. This now represents the most comprehensive licensing and inspectorate regime in the world under the auspices of the HFE Authority. Importantly, it has also served as something of a template for regulatory regimes that followed. For example, the UK instituted the labyrinthine Human Tissue Act 2004 with its associated licensing Authority as a direct result of the Bristol and Alder Hey organ retention scandals that arose from sub-optimal (or non-existent) consent practices relating to forensic and research uses of deceased children’s tissues. This regulatory endeavour replaced one legislative instrument of four sections with a new law 25 times its length.^3^ Arising in parallel have been European and other supranational influences. Thus, within the European Union, the UK and its 27 fellow Member States must comply with the new Clinical Trials Regulation,^4^ the Data Protection Directive with respect to data use for research (to be replaced by the General Data Protection Regulation),^5^ medical devices directives (also en route to Regulation status),^6^ and hybrid provisions such as Advanced Therapy Medicinal Products (ATMPs: similarly subject to centralised legislative reform).^7^ A multiplicity of regulators attach to these legislative structures creating not only a highly complex and tightly regulated landscape, but also an associated taxonomy of regulated ‘objects’ such as ‘data’, ‘tissue’, ‘embryos’, ‘devices’, ‘ATMPs’, and ‘clinical trials’, each bounded by its own legal definition and bespoke sets of regulatory rules of production, storage, use, and market approval.

This pattern of command-and-control regulation^8^ whereby a state authority has dominant oversight and power over health research is repeated throughout the western world and beyond. It is also often driven by legal instruments that adopt a similar ‘bounded object’ approach, that is, where law creates artificial constructs that become the object of regulatory attention of dedicated regulators who operate within legally defined spheres of influence or ‘silos’.^9^ At the risk of caricaturing the law in health research, the regulatory paradigm is nevertheless commonly typified by a central role for, and demand on, the law to establish and dictate regulatory practices in an increasingly silo-dependent manner. It is manifestly the case that health research is subject to closer scrutiny than at any time in human history. But where is the ‘human’ in this story? Why are the literatures and the media replete with concerns about a lack of trust in health research or uses of human materials and data *despite* this plethora of regulation?^10^

Most recently, the UK Care Act 2014 now requires eight leading regulators and government bodies to work together under the oversight of the Health Research Authority towards ‘co-ordinating and standardising practice relating to the regulation of such research’,^11^ while having regard for the need:

- to protect participants and potential participants in health or social care research and the general public by encouraging research that is safe and ethical, and
- to promote the interests of those participants and potential participants and the general public by facilitating the conduct of such research.^12^

The adoption of this latest piece of legislation is clear recognition that the research enterprise continues to operate sub-optimally despite considerable advances in human rights, ethical discourse, public engagement, and reflexive governance. This raises important questions about how the 2014 Act might actually deliver on its stated objectives. It is noticeable that recourse to law remains utterly central to the delivery of health research regimes.^13^ However, a central premise of this article is to suggest that the legally centric approach thus perpetuated is problematic.

The article proceeds as follows: Section II examines what the current construction of regulatory space in health research actually delivers, as driven by law. It offers the example of the *care.data* initiative involving unwarranted uses of citizens’ health data to suggest that there are many reasons to be wary of reliance on law to legitimise health research practices; Section III then develops an alternative analysis of these practices through the anthropological lens of liminality. It is argued that the human experiences within regulated spaces matter. Moreover, the literature on liminality helps us to think about those spaces in radically different ways—notably as processes of transformation and change—and with important consequences for health research regulation; Section IV offers a range of examples that show *why* this perspective matters; finally, Section V invites the reader to reconceive the research enterprise as involving liminal regulatory spaces, and the consequences of this are fully explored.

## II. WHAT IS THE RESEARCH ‘PROBLEM’ WITH HEALTH RESEARCH REGULATION?

It is a misnomer to talk about *the* regulatory space in health research. The ‘bounded object’^14^ approach to regulation mentioned in Section I has arisen partly because of piecemeal developments over the past 70 years, partly because of the diverse agenda of multiple national and supranational actors with authority to act, and partly because of serial knee-jerk reactions to moral panics, such as Alder Hey and Bristol. It has a number of unlooked-for consequences. First, it creates a series of legal silos where regulation occurs in largely disconnected ecosystems;^15^ thus, ‘data’ are subject to one regime while ‘tissues’ are subject to another, and ‘embryos’ to yet another, and so on. Secondly, and relatedly, this not only fails to reflect the reality of research practice – i.e. tissues are of value precisely because they reveal valuable data – but it also makes a mockery of any notion of a uniform and unitary regulatory space.^16^ There is, instead, a multiplicity of spaces ostensibly engaged in the same endeavour but with little means to learn lessons between them.^17^ Thirdly, this potentially militates against the efficiency and proportionality of regulatory oversight in complex research protocols, that is, when research straddles different regulatory regimes and involves multiple agents and regulators. Embryonic stem cell research is an example of this.^18^ Lastly, and most importantly, this approach makes the ‘object’ and not the ‘subject’ the primary focus of regulatory attention.^19^ That is, the experiences of the research participant qua being a (research) *subject* are neither fully captured nor necessarily accommodated by the regulatory regimes.^20^ While it is shown below that laws do, of course, seek to protect research participants’ rights and interests, it is often forgotten that there are other *subjects* involved in the research enterprise too, not least researchers and regulators. Yet, regulatory attention is neither overtly nor systematically directed at their experiences, and we must ask if something is lost as a result.

As suggested immediately above, it is undeniable that serious efforts have been made to put research participants more at the heart of the research enterprise. The most obvious example of this is the primacy of consent in today’s legal research regimes. There is, nonetheless, a disjunct between consent that operates as a quasi-legalistic waiver of rights akin to the one-off signing of a contract, and consent as a *process* through which participants might be able to exercise continuing control.^21^ Albeit that there has been discussion in the literature about consent as process,^22^ this still suggests that consent operates in a largely instrumental fashion. There remains a serious lack of understanding about what experiences these appeals to consent represent, or even mask, in terms of what it is like to be—or to *become*—a research participant.

Additionally, the consent paradigm is largely individualistic in outlook, especially as established within human rights frameworks focussing on rights and interests. This sits at odds with the objectives of many health research regimes that are community-focused and usually premised on some form of altruism in terms of what they expect of participants. Indeed, Thomassen and others remind us that the term ‘interests’ stems from the Latin words ‘*inter*’ and ‘*esse*’: in-betweenness as between beings. The notion of interests refers to that which lies between, or happens between, beings or being: ‘it is a “being in between”.’^23^ According to Thomassen, and following Girard,^24^ ‘… what we want, what we desire, happens between human beings. The value of an object lies not in its intrinsic qualities, but in how that object is viewed by more than one subject’.^25^ This implies that neither the notion of the rights holder nor the focus on the bounded regulatory object fully or accurately capture what is happening in human health research. It suggests that our current approach to human health research is ‘… inherently problematic because it fails to treat the anthropological dimension in any serious way.’^26^

In contrast to what is said above about diverse regulatory silos, there are nonetheless examples across regimes of similar or common *approaches* to regulation. An example in the data and tissue regimes has been the emergence of the so-called ‘consent or anonymise’ approach. This adopts a binary either/or model of regulation whereby the protection of research participants’ rights and interests is considered to be satisfied either by seeking consent to data/tissue use, or by anonymising data/tissue to minimise the likelihood of identifiability (and so reduce threats to privacy). This regulatory approach has been subjected to sustained critique in other contexts both for its failure effectively to protect research subjects,^27^ and for the limiting effects it can have on the execution of robust research.^28^ The novel point for present purposes is, however, with respect to the repetition of the approach across regulatory spheres: it suggests that there can be a tendency across regimes to reproduce or *mimic* regulatory responses without due attention as to whether a regulatory device that is fit for one context is also appropriate in another.

Taken together, these observations suggest that, despite the central role of law in driving research practices, law’s regimes do not capture the experience of *becoming someone involved in research.* Thus, when we ask the earlier question, where is the ‘human’ in this story about human health regulation?, we currently receive an incomplete answer. This is worrying and unacceptable because, from the human perspective, the process of becoming a research participant is a change of status, potentially in very profound ways.^29^ Individuals, their bodies, body parts, and other intimate adjuncts such as personal data, are instrumentalised to varying degrees. The core question, then, is: if we see involvement in research as a change in status as human beings, what might that mean for our understandings of health research regulation?

A recent example of regulatory failure helps to bring all of this into stark relief and strongly suggests that a new focus on the human experience of being involved in health research is both vitally important and urgently required.

### A. care.data: Where is the ‘human’ in Human Health Research?

The *care.data* initiative was introduced in England under the umbrella of the Health and Social Care Act 2012 which set up the Health and Social Care Information Centre (HSCIC) to receive individual level patient data from GP surgeries. The aim was to deliver six key commissioning and service objectives, including ‘economic growth, by making England the default location for world-class health services research’.^30^ In a version of the ‘consent or anonymise’ paradigm, patients were not asked for consent, and instead data would be used in anonymised form as the default.^31^ Where this was not possible, approval was to be sought from the Confidentiality Advisory Group, also operating under legal authority,^32^ to authorise identifiable, non-consented uses ‘in the public interest’.^33^ Furthermore, the NHS Constitution was amended in 2012 to provide for the initiative, notably through restatement of the claim that it served a public interest, and a ‘pledge’ to inform NHS patients about research studies for which they might be eligible. Leaflets were sent to 26.5 million households, although not addressed to individuals qua patients; moreover, this information contained no direct mention of *care.data*.

Within weeks of its launch *care.data* was suspended in February 2014 amid vociferous patient and professional objections about lack of transparency and robust oversight, as well as concern about inadequate information on possible uses and disclosure of data, especially to commercial entities. Elsewhere, it has been suggested that a partial explanation for this reaction is a lack of ‘social licence’, ie the assumption of a mandate for action must accord with the general expectations of society, and it is dangerous to attempt to borrow this wholesale from one context to another.^34^ In the case of *care.data,* the trust within the GP-patient relationship and the basis upon which health research is otherwise conducted could not be assumed to translate to a top-down, faceless, bureaucratic enterprise.

Notwithstanding, even if this explanation holds true, it only provides a partial picture because it addresses only a community-based response. To be more complete, we require *both* an individual-level and a community-level human experiential explanation. It is in this respect that the anthropological concept of liminality can greatly deepen our understanding, not only of an initiative like *care.data* but also of the dynamics of human health research more generally.

## III. LIMINALITY

The concepts of ‘liminality’ and ‘liminal space’ were first developed by the anthropologist Arnold van Gennep in his seminal work, *Rites of Passage*, published in French in 1908. In a veritable *tour de force* across human history and cultures, van Gennep posited that it is a universal constant when human beings transition from one social status to another—for example, from childhood to adulthood, singledom to married life, and even life to death—that a *pattern* always occurs that involves three distinct stages: 

… the [i] rites of separation from a previous world, *preliminal rites*, those executed during the [ii] transitional stage *liminal* (or *threshold*) rites, and the [iii] ceremonies of incorporation into the new world *post-liminal rites*.^35^

Van Gennep was interested in the form of this schema,^36^ and notably with the *liminal*—the in-between—stage, which acquires ‘… a certain autonomy’.^37^ The term derives from the Latin for threshold, ‘limen’. It signifies the crossing of a threshold and a *change* of status for the person concerned. Moreover, as van Gennep observed: ‘[s]uch changes of condition do not occur without disturbing the life of society and the individual, and it is the function of rites of passage to reduce their harmful effects.’^38^

Thus, the importance of rites of passage emerges as a means to manage transitions through liminal periods and spaces. A central feature of such processes is the role of an independent actor to guide those persons experiencing liminality through the liminal phase and out the other side. This role has been referred to variously as a Master of Ceremonies^39^ or the Representative of Order.^40^ Stenner and Moreno-Gabriel point out why this is so important in terms of the subjective experiences of the people concerned:

Moments of “becoming” are literally formative experiences in which subjectivity itself acquires a new pattern… any social artifice of staging does not contradict the authenticity of experience: the more important and emotionally real the event, the greater the need for a carefully staged structure to “occasion” the experiences. In the absence of such patterning devices, liminality can be chaotic, messy, dangerous and destructive. Instead of being a formative experience, emotional transitions can be de-forming.^41^

Albeit that van Gennep developed his work on liminality in the context of tribal societies,^42^ its enduring relevance to modern mass societies has been forcefully and convincingly argued by a number of key contributors since the 1960s,^43^ when van Gennep’s work was translated into English and picked up by British anthropologist Victor Turner.^44^ One of Turner’s key contributions to liminality, set within the culturally transformative late 1960s, was to suggest that in liminal spaces people are released from social structures, and *anti-structure* takes over. As he described it:

The attributes of liminality or of liminal personae (‘threshold people’) are necessarily ambiguous, since this condition and these people elude or slip through the network of classifications that normally locate states and positions in cultural space. Liminal entities are neither here nor there; they are betwixt and between the positions assigned and arrayed by law, custom, convention, and ceremonial.^45^

For Turner,^46^ and others,^47^ the liminal space can be a moment of opportunity where pre-existing norms and constraints no longer apply. In particular, prior formal structure is often replaced by a sense of *communitas* among persons in a liminal state:

… [A]t certain life crises such as adolescence, the attainment of elderhood, and death, varying in significance from culture to culture, the passage from one structural status to another may be accompanied by a strong sense of ‘humankindness’, a sense of the generic bond between all members of society – even in some case transcending tribal or national boundaries – regardless of their subgroup affiliations or incumbency of structural positions.^48^

We return to the significance of this below.

For sociologist Arpad Szakolczai, van Gennep’s work on rites of passage and Turner’s insights about structure and anti-structure are profound:

Rites of passage have a simple and clear but by no means trivial structure, according to van Gennep and Turner, who draw on evidence from practically all the cultures of the planet. The core substance of Turner’s proposition is that this structure is the key to the structure of human experience.^49^

The crucial point is that, when considered objectively as perspectives on human experiences, the claim in much of the anthropology literature is that liminality reveals something *universal* about the human condition. This article does not suggest that any such omnipresent truth exists, nor does it seek evidence of this; rather it deploys this ‘myth’^50^ about the human experience to cast new light on the roles and limits of law in health research. It is on this basis that we can consider what liminality might teach about the medico-legal world.

### A. Liminality in the Medico-Legal World

An easily recognised example of where the myth of liminality helps us to understand the medico-legal context is the well-developed concept of the ‘mature minor’. This notion was first laid down by the House of Lords in the famous case of *Gillick v West Norfolk & Wisbeck Area Health Authority*.^51^ The essential legal dilemma was this: to what extent, if at all, ought the law recognise the growing autonomy of a child as she or he nears adulthood and with respect to intimate decisions about their own bodies? In this particular case, could a doctor lawfully prescribe contraception to a teenage child without informing the parents or seeking their consent? In developing the notion of the ‘mature minor’, the House of Lords offered a qualified Yes in answer to this question. The concept is subject to a five-point test the details of which are not necessary for present purposes because the overall approach is largely framed by: (i) sufficient maturity by the child to understand the nature and consequences of what was proposed, *and* (ii) an assessment by the relevant health care professional that the intervention and independent act was in the child’s own best interests. Thus, the mature minor is *neither* a child to be treated wholly in her own best interests, *nor* an autonomous adult at liberty to choose or refuse medical care at will. Indeed, it has frequently been confirmed that refusal by a mature minor can be overridden by responsible parents or the courts,^52^ in contrast to the absolute right to refuse of competent adults.^53^

The present analysis strongly suggests that the young person in such cases is experiencing a liminal phase of their life; they are literally passing through a temporary state of being where established legal regimes are disrupted and called into question. Law’s attempt to construct the status of the mature minor is, in turn, an attempt to give effect to the liminal experience. The value of this way of understanding what is happening has been largely lost on the majority of critics of the *Gillick* case. Many of the criticisms suggest that the House of Lords failed to recognise a binary consent-refusal power that apparently emerges in an instant.^54^ Liminality offers a very different explanation. Crucially, liminality is normally a transitory occasion and characterised by uncertainty. This helps to explain why law and society often struggle with how to deal with the mature minor.^55^ The explanatory power of liminality is well illustrated by this example. Key features are: (i) the focus on the experience of the subject as *between* states of being, (ii) the acknowledgement of the temporary nature of the experience—the person is passing *through—*as well as the transformative nature of the experience, at least in terms of legal status,^56^ and (iii) by implication, the attention is on the *process* itself.

In other health-related contexts, the potential value of insights about liminality has been recognised by various non-legal social science disciplines, notably by researchers seeking to understand the experience of disease-onset,^57^ ill-health,^58^ survival beyond disease, ^59^ and even with respect to end of life and palliative care.^60^ For example, engaging the group-based *communitas* idea spoken of above by Edith Turner, Froggatt has argued that:

[ T]he hospice movement … by creating an alternative place to die outside the dominant health care system, could also be considered to have established metaphorically liminal institutions where communitas may be experienced.^61^

However, what we do not have to date is a deep analysis of the value of liminality in understanding research participation, nor of the role of law in support of, or in facilitating, such practices.

### B. Deepening the Analytical Potential of Liminality

In offering such an enquiry, this article reflects the work of Thomassen who has recently written on liminality in modern society.^62^ He encourages us to recognise liminality and liminal spaces where they occur and for their shared properties, and to grasp the fuller and deeper meaning of their existence, with all that this entails.^63^ Thomassen highlights, in particular, that liminality has both temporal and spatial elements. Thus, for example, it can exist as a mere moment, over a sustained period, or into epochs, and it can occur in specific places or with respect to specific objects, including the human body. It can occur in closed areas or institutions, and in larger territories within, and even beyond, entire countries. In all cases, it can be experienced as much by individuals, as by groups, and/or by societies at large. As an example of the last, Thomassen offers the 18th century Lisbon earthquake, which was a natural disaster of such tremendous proportions that it led to a rupture with old European ways of thinking about the world, necessitating radical reforms of urban planning and renewed humility of man about his own progress and attempts at dominion over nature.^64^ The same might be true of the global threat from pandemics today.^65^ Such an analysis also helps us to understand socio-legal responses to medico-moral panics as processes that almost *demand* change and transformation from previously prevailing circumstances. This is particularly important for the law, because the law is often called-upon to lead society out of a particular crisis. We have seen this all too often in the health research arena, as Section I of this article demonstrates. But even if this is so, what does the recognition of the existence of liminality tell us about the *process* of moving forward—either as individuals or as a collective? In particular, how can it be that liminality can encompass both moments of opportunity (Turner) and moments of crisis (Thomassen)?

Thomassen suggests that here we require further anthropological concepts to reveal the ‘analytical potential of liminality’.^66^ Building on Szakolczai,^67^ he offers three that can be characterised as:

- *imitation* (mimesis),
- *deception* (trickster), and
- *division* (schismogenesis).

The claim is that the uncertain and ambiguous nature of a liminal state of affairs exposes three particular dangers: (i) a tendency towards unreflexive imitation and imitative behaviour or patterns in the absence of a clear path to follow out of liminality;^68^ (ii) a risk that a ‘shaman of the liminal’—a trickster—will present themselves to exploit the situation, claiming to lead the way to salvation but for nefarious ends:^69^ this is an important counterpoint in mass societies to how liminality presents in tribal societies—as indicated above, normally rites of passage have a Master of Ceremonies to guide initiands out of the liminal void but if there is no obvious Master of Ceremonies, the risk of exploitation is heightened; and (iii) there is a final risk that the transformative process will not be completed because of the (negative) cumulative interaction between parties: this can result in either a state of permanent liminality where a state of crisis is perpetuated, or the incorporation of a ‘schism’ into society itself at the re-integration phase and a ‘splitting off’ of groups.^70^

This suggests that liminality can produce both negative and positive group-based experiences. The risk of ‘schism’ is clearly negative and could signal the end of any well-intentioned legal or regulatory plan for society. In stark contrast, Victor Turner’s identification of *communitas*, already mentioned above,^71^ offers an explanation of an altogether more positive experience. Different both to the idea of community^72^ or even solidarity,^73^ communitas refers to a spontaneous sense of interconnectedness of equals, experiencing the same process together: ‘[i]t is … a matter of giving recognition to an essential and generic human bond, without which there could be no society’.^74^ As Stoller has said:

People in liminal states tend to be humble. They usually do what they are told – often without complaint. They accept regimes of pain. They are reduced to a common denominator so that they might be reconstructed. These processes create an intense camaraderie, which washes away difference in age, social status, and ethnicity. Turner called this camaraderie ‘communitas’.^75^

The immediate parallels with patients suffering a common disease, or of patient groups rallying to collective action, should be obvious.^76^ However, as Edith Turner points out of *communitas*, it is characterised by ‘ … its shyness and untouchability by commercialization and institutionalization’.^77^ Communitas is powerful for its ability to bind human beings through common experience, and its potential power is all the stronger for its emergence in liminal spaces where existing social structures, hierarchies, power dynamics, and established institutions are called into question. Yet, at the same time, this anti-structural and ethereal happening is entirely dependent on the spatial and temporal limits of liminality itself. As she comments:

Communitas is exciting; it makes people able to organize and work together. With this power, they will eventually develop organizational habits, structures, and rules of behavior, and ranks and positions. These often work well if they remain on the human level; yet if they become overly law-bound, communitas will bubble up again from below and question the old system.^78^

Communitas therefore defies design and manufacture. It cannot be created artificially. Moreover, this suggests important limitations on regulatory or legal regimes that attempt to prescribe too closely the dynamics of human experience. This is not to say, however, that liminal spaces cannot be facilitated for their productive potential, so long as this is on the understanding that that they are likely then to assume a dynamic of their own. Examples of this in research and policy terms might be the Sandpit Methods of early involvement in research and technology design^79^ which involve ‘ … intensive discussion forums where free thinking is encouraged to delve into the problems on the agenda to uncover innovative solutions’.^80^ As for liminality itself, however, it is important to recall that Edith Turner’s analysis suggests that it is not amenable to regulatory control or influence in the same way as other regulatory approaches.^81^ Where, then, does this leave us?

To recap, an analytical framework of liminality appears to have the following features:

- First and foremost, it is *experiential;*
- It is both potentially *transformative* and, normally, *transitory;*
- It is *processual,* having both *spatial* and *temporal* dimensions;
- It is characterised by *uncertainty* and has considerable disruptive potential;
- It is *anti-structural* in the sense of challenging existing and established structures;^82^
- *Rites of passage* therefore have value in guiding people through liminality;
- *Independent actors* can also assume a valuable role as guides through liminality;^83^
- Where rituals and actors are absent there is a greater risk of chaos and attenuated uncertainty or permanent liminality;
- Other risks include unreflective *mimic* behavior, the rise of the *trickster*, and perpetual *schism*, i.e. a failure to complete the process and achieve social re-integration;
- Ultimately, to pass through liminality is often to experience a change of status;
- This can be accompanied by the emergence of *communitas* by the persons involved, but this is spontaneous and cannot be fabricated;
- Liminality can, then, be both challenging *and* empowering, but it is not easily amenable to direct influence or control.

As a final point, we must ask how—if at all—we can reconcile seemingly positive experiences of liminality (Turner) with negative ones (Thomassen)? There is no definitive answer, but a partial response seems to lie in the *context* for the creation of liminality—did this arise from a crisis or from established ‘ritual’ process? Another important feature turns on the *actors* involved—who guides us through liminality and with which intentions? All of these features have potential implications for health research regulation.

## IV. LIMINALITY IN HEALTH RESEARCH REGULATION

These insights allow us to ask profound questions about the experiences of being involved in health research regulation and hold real potential to reveal new understandings about the serious challenges that persist in delivering acceptable and effective regulation. Fundamentally, we can enquire: are there liminal spaces *within* the health research regulatory space(s)? How can we recognise them? Indeed, who—or what—is in a liminal space, and what does it mean to experience it? Finally, where does this leave us on law, policy, and practice?

### A. Are there Liminal Spaces within Health Research Regulation?

There are many features of health research that suggest that *multiple* liminal spaces exist within the regulatory environment, both for the human beings involved, and also with respect to the objects that are so central to the regulatory enterprise.

#### 1. Consent as a Liminal Process

As to the human experience, consider the practice of research recruitment. In the paradigmatic example of the clinical trial, the obtaining of consent takes pride of place. This is a highly regulated and proscribed procedure in most legal systems, dictated as to its form and function by legal architectures, such as the European Clinical Trials Regulation,^84^ and the specific guidance of research ethics committees.^85^ Indeed, we can see the consent process itself as a form of *ritual* involving established roles and formalism—not least the signing of the consent form itself—designed to initiate persons into a new social context. As Wilson has suggested:

Rituals reveal values at their deepest level … men express in ritual what moves them the most, and since the form of expression is conventionalized [sic] and obligatory, it is the values of the group that are revealed. I see in the study of rituals the key to an understanding of the essential constitution of human societies.^86^

The values underpinning the consent process have been well rehearsed elsewhere.^87^ Seeing it as a ritual, however, helps us to understand consent as a potentially *transformative* experience. In stark contrast to the ‘consent moment’ being a mere quasi-contractual waiver of legal rights completed with the signing of the consent form, this also adds meaning to talk about consent as *process*^88^ and requires us to consider the multiple dimensions of such a process. From the experiential perspective, the process is one of change of status for the persons involved: *from* patient *to* research participant; *from* healthy volunteer *to* community contributor etc. Moreover, this happens outside law and in some sense is pre-law: no change in legal status occurs. Nonetheless, liminality requires us to take this transformation and these multiple subjectivities seriously. We can begin to do so by following through the entire process: having crossed the threshold of giving consent, we should ask, what happens next and what unmet needs still require regulatory attention? This arises as a consequence of creating a liminal space for research participants by obtaining their consent to research.

In terms of transformation, liminality can call into question people’s own identities. Being recruited to health research means that people are no longer *just* a patient or ordinary citizen; they acquire a new and additional status of ‘research participant’. Moreover, they are called upon to give something of themselves, whether this be simply their time, or also personal information and/or physical parts of their bodies, including blood, DNA, tissue samples, and sometimes whole organs. Health research requires participants to ‘actualise a new cross-domain identity’.^89^ They must do so in a context and environment that are necessarily uncertain—this is the very definition of why research is necessary and justifiable: it is about generating new knowledge from the unknown.

More specifically, liminality focuses our attention on the temporal and spatial dimensions of this experience. For example, it leads us to ask: for how long does the liminal phase last and what lies beyond it? While this might be a difficult or impossible question to answer because the timeframe will often be indeterminate, it is certainly longer that the ‘consent moment’ itself, ie the mere signing of the consent form. Indeed, a fixation on consent as ‘the’ defining moment in health research is misleading if this leads us to ignore the longer-term experiential dimensions of becoming a research participant. Moreover, liminality and its accompanying uncertainty and disruption should lead us to question whether mere voluntariness of participation is sufficient to protect and support research participants.^90^ The consent form might be an important initial element of the rite of passage into the realm of research, but it should signal the beginning—the threshold—of a change of status, not an end point in itself. The subsequent transition and uncertainty are likely to leave research participants ill-equipped to navigate the new territory alone. Thus, as Ladge and others have argued: ‘Liminality is not just a matter of knowledge and cannot be “informed away”, as it is germane to socio-cultural processes and structures.’^91^

Rather, liminality suggests the likely importance of a role for a Master of Ceremonies or Representative of Order to guide people through. We do find examples of these kinds of roles in, for example, cohort studies that have dedicated teams to liaise with cohort participants and to manage research projects involving cohort members.^92^ A paradigm example is the Children of the 90s project, which has ‘[s]ince the outset, … had [its] own ethics committee, which comprises clinicians, researchers, lawyers and lay people, including study participants’. As the project states on its website: ‘[w]e involve participants in many aspects of the study and they have their own advisory panels and online discussion forums’.^93^

A liminal perspective, then, suggests much more of a need to develop and support ideas about authentic research partnerships between researchers and participants alike.

#### 2. Research as a Liminal Process

In acknowledging that liminality re-orients our attention towards the inter-subjectivities at play, this should then also be extended to include interactions not just between research subjects, but also between and with researchers and regulators too. While it may not be the case that researchers are transformed in status terms, their entry into a liminal space of uncertainty—ie the research endeavour—still suggests the value of a role for a guide. For example, the Health Research Authority has recently appointed officers known as Applications Managers to help guide researchers through ‘complex cases’ that straddle regulatory regimes.^94^ Equally, in the data linkage for research domain, such as that represented by the Scottish Health Informatics Programme,^95^ Research Coordinators work with researchers to refine research questions and broker discussions with data controllers whose data are sought.^96^ These examples are in contrast to how many research ethics committees operate in most health research contexts. They serve an important gatekeeper role only at the principal threshold at the initial outer limit of the research landscape to ensure that robust up-front ethical scrutiny is conducted. However, they are rarely involved in, or empowered to, accompany researchers and participants through the research process itself. However, if we see the research endeavour itself as a liminal process, then there are many points of transition and transformation—multiple thresholds—of which initial ethics approval is only a beginning. Other thresholds include protocol deployment, generation and capture of research findings, publication and dissemination of research results, and data storage and sharing for future research. As a result, core concepts to which appeals are often made in this context, such as the ‘social value’ of research, are potentially transformed multiple times and across multiple spaces occupied by a range of (potentially constantly changing) actors.^97^ This contributes to the difficult of adequately defining such concepts. Liminality allows us to engage with this fluidity and requires that we pay attention to what happens in these spaces in-between.

For example, Stephens and others have provided evidence of what they term *instantiated* regulation emerging in relation to the operation of the UK Stem Cell Bank. Their fieldwork has revealed the emergence of ‘embedded regulators’ in everyday laboratory practice in order to make regulatory documents ‘do-able’. That is, while regulations and procedures prescribe certain conduct in order to conduct the research, the rigid following of the letter-of-the-law actually hinders the research. The regulation is, therefore, managed by the actors on the ground. The suggestion is that in this realm there is a tension between (legal) text and (practical) context in giving effect to regulatory prescription for the ‘doing’ of research. The authors argue that ‘instantiated regulation’ emerges as ‘…a response to the interpretative flexibility of regulatory texts’.^98^ To add an interpretation from the current analysis, this further suggests that the liminal research space that is represented by the UK Stem Cell Bank requires actors to lead researchers through the space if the research and the overall objective are to be achieved. There is a role here, then, for regulatory *stewardship*.^99^ This is not something that is currently captured by formal legal regimes. It is a phenomenon that is likely to be revealed by the lens of liminality.

#### 3. Regulated Objects as Liminal Entities

Finally, and as stated above in sub-Section IV A.1, involvement in health research usually requires participants to give of themselves, be this time, data, and/or tissue. The separation of elements of self can represent a form of crisis, necessitating new ‘forms of process’^100^ to deal with what occurs. The crisis is often seen as new threats to privacy, or to a lesser extent to identity, through loss of control. This is, in fact, where law is most often called upon to intervene. The myth of liminality can once again assist our understanding here when we appreciate that liminality might not only experienced by *persons*. The ‘bounded objects’^101^ of health regulation described in Sections I and II above—‘data’, ‘tissues’, embryos’ etc.—can also be seen in these terms. Squier, for example, has argued that supernumerary embryos from artificial reproduction technologies represent ‘liminal lives’ that exist in ‘… that in-between or marginal zone … neither discarded by-product nor valued human being, they are participants in a rite of passage, between everyday life and a higher or different level of existence’.^102^ We can view ‘personal data’ and ‘human tissue’ in similar terms, and as defined respectively by the Data Protection Act 1998 and the Human Tissue Act 2004. This is because what counts as ‘*personal* data’ or ‘*human* tissue’ can shift and change over time depending on contexts for storage and use, and importantly, whether living human subjects are identifiable from the data and tissue in question at any given moment. Thus, when persons are identifiable from data or tissue that are used or stored, these materials are imbued with a specific significance and regulated as such by the respective pieces of legislation. If that identifiable link is broken, the laws cease to have effect. It is often forgotten, however, that such links can be re-established over time, as when the holder of anonymised data comes into possession of new data that renders the subject identifiable once more.

Both the 1998 Act and the 2004 Act explicitly recognise the connection between regulatory objects and the human subjects from whom they are derived. In both systems, consent is again a principal legitimating factor in dealings with the objects in question. Equally, both deploy anonymisation as a mechanism to attempt to protect the subjects from particular uses or dealings with the data or tissue that have been extracted. Thus, as noted previously, the ‘consent or anonymise’ paradigm is replicated—or mimicked—across both systems. This mimetic behaviour is also predicted by liminality, which suggests that copying in times of crisis is a common human response. One such time of crisis involved the Bristol and Alder Hey scandals in the 1990s in which lay the origins of the Human Tissue Act.^103^ The ‘consent or anonymise’ paradigm was already a well-accepted practice under the data protection regime and it also found it way into the 2004 legislative response to the crisis around human tissue.^104^

The added value of an analysis grounded in liminality is that it leads us to question more generally the adequacy of these legalistic responses. Comments have already been offered as to the limits of consent. As for anonymisation, the irony is that the attempt to break connections with individual research participants perpetuates uncertain liminal states, leading to permanent liminality.^105^ There has been much discussion in the literature of the inadequacy of anonymisation techniques,^106^ and many calls to recognise that identifiability is a relative and contingent term,^107^ depending not only on techniques applied initially to data or samples but also subject to change depending on new information or connections made *over time*. Thus, for the purpose of this article if we were to combine a spatial and temporal analysis of anonymisation—involving fragmentation of self and uncertain future possibilities—it leads us to understand anonymisation as a process occurring in a liminal space with respect to data and/or tissue. Moreover, this analysis can help to explain ongoing anxiety about data/tissue uses removed from persons.

In its attempt to create ‘bounded objects’ as the appropriate focus for regulatory attention, the law seems to overlook the experience for the data or tissue *subject*. An alternative regulatory perspective would be to recognise and acknowledge the enduring connection between subject-object, and the potential identity-significant implications of this. This does not necessarily mean that research participants be granted a continuing control right over their data or tissue,^108^ nor does it mean that anonymisation becomes a redundant security measure. Rather, it suggests that more attention should be directed to addressing participants’ expectations when they contribute to the research endeavour. This might include, for example, the deployment of traceability mechanisms to alert participants to particular uses of their contributions. An example of this is the sms-text system used in Sweden to inform donors when their blood has been used.^109^ In the same way that the person’s status undergoes transformation during health research, so too do their donations. Arguably, the expectation cannot be that tissue/data remain unchanged. Rather, these entities become a valuable research resource to be used for the public good. Due acknowledgement of the achievement of such an objective at the level of one’s own participation in research could go a long way to promoting genuinely communal interests: *between* beings.

### B. *What doesLiminalityRevealAbout care.data?*

Let us return to the events surrounding *care.data*, as outlined in Section II, to bring this analysis together. What does liminality reveal about what occurred? First, and perhaps most tellingly, it was law itself that created the liminal space. It will be recalled that the Health and Social Care Act 2012 established the Health and Social Care Information Centre with legal authority to require patient data from GP surgeries. Neither GPs nor patients were actively involved in assenting to these processes. Rather, the legal regime mimicked a version of the ‘consent or anonymise’ paradigm. The likely effects on patients of being thrust into a new status of research ‘subjects’^110^ have been addressed above, but what of the GPs? They too were required to ‘actualise a new cross-domain identity’^111^—moving from a status of primary care provider to acting also as agent of the state concerned with a far wider range of considerations than simply patient health. This raises the prospect of the role of ‘trickster’ in liminal terminology. Liminal spaces can be dangerous not only because they create uncertainties and disturb established social structures, but also because they leave the people who occupy those spaces vulnerable to deception and *mis*-leading actors. In contrast to the Master of Ceremonies or the Representative of Order, the trickster does not act as an independent guide through liminal space; rather he acts for his own interests, or at least not in the interests of those experiencing liminality.^112^ Liminality suggests that there was a double bind operating with *care.data*. From the patient perspective, it cast suspicion on GPs as possible tricksters; and from the GPs’ perspective, who also experienced liminality, they were left without a guide through the new liminal space. Indeed, we might even suggest here that law itself was the trickster by creating a liminal space and subsequent crisis and for failing to provide suitable rites of passage through the new landscape. In this example, law led people into liminality, not out of it. It provoked crisis, rather than resolved it.

A further observation follows from this. It would neither be a sufficient nor complete response simply to better inform patients and GPs, nor indeed to secure their consent to such an enterprise. While there are strong ethical imperatives to suggest that this ought to happen *as a minimum*, the creation of liminality with respect to patient data use and GP practice requires a longer-term, supported, guided, and experience-focused plan of action if an initiative such as *care.data* has a chance to succeed. If this does not happen then the third danger of liminality—division—is likely to prevail. The schism in question here could occur at multiple levels: patients with respect to GPs; GPs with respect to employers and the NHS; researchers with respect to regulators; and so on. Moreover, the failure to recognise the ongoing subject-object relationship, and to address associate expectations, is equally likely to signal failure for any re-launch of *care.data*. Liminality thus throws into stark relief the reasons why the mere rendering of processes as ‘lawful’ is inadequate in, and of, itself.

The subsequent plan of action for *care.data* can be assessed in such terms. In May 2014, the *care.data* Programme Board agreed to work with up to 500 practice ‘Pathfinders’ to test, evaluate and refine all *care.data* technical and communication processes.^113^ The Programme adopted a ‘co-production’ approach to initial GP and patient-facing material. Moreover, opt-out was then clearly embedded for all citizens to be approached. The Programme further identified a list of issues considered to be essential to demonstrate the success of the Pathfinder stage,^114^ as well as a further set of questions required to be addressed adequately. Central to this were questions from GPs about what support would be available to ensure they discharged their duties. The preceding analysis would suggest that this plea to be guided through is of particular importance. There is some irony, in fact, that the initiative was called Path*finder* since the path through this new regulatory space is far from clear. Furthermore, from a citizens’ perspective, there remained questions about the transformative potential of their new status as citizen contributors. Liminality would suggest that more express consideration be given to what this might mean and how citizens experience this. This might include, for example, ongoing engagement and feedback of benefits as part of a *process* of involvement (rather than simply the co-production of up-front information). Indeed, at the time of going to press, two reports were published recommending a consent/opt-out approach to data sharing in NHS England.^115^ On the same day the Department of Health and George Freeman MP announced that the *care.data* programme would be closed.^116^ Public consultation on the proposed approach would follow.

### C. *What doesLiminality not do?*

It is important to offer some caveats about the limits of this analysis through the lens of liminality. This is not an empirically grounded study. Thus, to reiterate there is no assertion that patients, GPs, or other actors actually or always experience uncertainty or anxiety with respect to health research. Nor is it crucial to the value of the analysis to claim that these experiences *necessarily* must occur before liminality can provide useful insights. Rather, an understanding of liminal spaces and liminal experience has a reorienting effect on our perspectives about what it means to be involved in health research. We must also recall both the perpetual and the ethereal nature of the myth of liminality. This suggests that any regulatory response is doomed if this is not taken into account, as the final section discusses below.

## V. IMAGINING *LIMINAL* REGULATORY SPACES

The remaining task is to consider how we might reimagine regulatory spaces in health research through the lens of liminality, and to ask what might be lost when we fail to pay attention to the spaces in-between. Accordingly, we can revisit the characterisation of legal regulatory space given in Section II above, as follows:

1. The attention of law and regulation on ‘bounded objects’ such as personal data and tissue should be questioned on at least two counts: first, for the fallacy of attempting to ‘fix’ such regulatory objects, and to divorce them from their source and the potential impact on identity for the subjects themselves; and, second, for the failure to see such objects as also experiencing liminality. Both of these points suggest a need to approach the management of regulation in processual and potentially transformative terms.
2. There is a plurality of research ‘subjects’ that experience the transitoriness and uncertainty of the liminal spaces of health research regulation. Thus, while it is fit and proper that due attention be paid to research participants and their experiences (including the status change that they undergo when law and practice convert them from mere citizens to agents of the public good), there is also a need to avert to the impact on researchers and regulators, especially if they are thrust into new regulatory spaces by state or institutional policies.
3. Equally, the primacy of consent as an individualistic concern should be challenged for its failure to provide complete protection or recognition of the full range of interests at stake within liminal experience. In particular, those interests extend beyond atomistic individuals. Given the socially-oriented nature of much health research, we should also consider the likely liminality experienced by the patient groups, communities, and publics more widely generated by health research as a public enterprise. If citizens experience this as an apparently haphazard, unguided, and potentially deceptive encounter, then liminality suggests a chaotic response will ensue. In contrast, it should be recognised that liminal spaces can be created to support the active involvement of groups in effective research design and regulation. Where *communitas* might arise—which is by no means guaranteed—this can have a particularly powerful influence, but the temptation should be resisted to attempt to manufacture it by design.
4. The role of consent as a legitimating event in health research regulation should be recognised as merely a threshold moment at the beginning of an attenuated experience. Consent should be re-envisioned as a process that takes on the form of *ritual*; there is a need to pay more attention to the transformation of status that consent signals and to provide more support through the process of experiencing involvement in health research regulation.
5. In addition, regulatory regimes should be alert to the risks associated with liminal spaces. The common regulatory response of ‘Consent or Anonymise’ can be seen as an example of mimicry when faced with crisis; equally, too, law’s role in provoking crisis (Trickster) rather than resolving crisis (Representative of Order) must be carefully examined. The failure to recognise these risks can eventually lead to schism within relevant communities, and a state of permanent liminality, uncertainty, and distrust. Thus, in this sense, an analysis of liminality also explains what is at stake in the perpetual pursuit of the public’s trust in health research regulation.

Ultimately, more attention should be paid to *process* and *transformation* within the regulatory spaces of health research regulation. If we return to the origins of the discussion of liminality, and the phenomenon first identified by van Gennep, we are reminded that liminality is the middle process of a three-part *pattern* of experience: (i) separation from existing order, (ii) liminality, and (iii) re-integration into a new world. The aim is completion of this tri-partite process. And this begs the question: what might be the New World in health research regulation? In his discussion of Liminality and The Modern, the answer for Thomassen is to return to stability and normality:

This happens by forging a new identity in the individual case, reflecting shift of one’s position within the social order; while in the case of society new common bonds are formed through the cathartic experience of communitas.^117^ Both processes involve the social and the asocial, and re-draw the boundaries between them.^118^

Put otherwise, the objective should be to bring people successfully out of the other side of liminality. If, then, we recognise and accept the disruptive potential of health research as creating liminal spaces—including through the law itself—then the responsibility is to consider what can be done to support people through liminal periods. And as emphasised in Section IV, this applies as much to researchers and regulators as to research participants.^119^ Self-evidently, there is no single answer to what all of this means across the vast range of research activity. Rather the triadic analysis requires serious reflection on when and where re-integration occurs and what this looks like. If we are not clear about this part of the process and do not support it adequately, then uncertainty is likely to endure; in turn, it is likely to foster distrust and other negative consequences.

Fruitful avenues to explore include the enduring appeal of *ritual* in contemporary society. We can, therefore, distinguish between two forms of liminality:

- Ceremonial liminality that is subject to rites of passage that guide people through liminal spaces or time periods, and which lend themselves to an independent representative of order; and
- Spontaneous liminality, which is unforeseen and resulting from crisis.^120^

Effective regulatory mechanisms need to envisage both of these eventualities, and to plan accordingly. The respective strategies must, necessarily, be quite distinct. The crucial reflective point for stakeholders accordingly becomes: how do current regulatory apparatuses structure process and transformation, and what are the benefits and shortcomings of this? We could consider, for example, the recent addition in the Care Act 2014, mentioned above, which requires the Health Research Authority (HRA) to appoint a committee for the purpose of giving advice to the HRA itself, the Secretary of State, and the Health and Social Care Information Centre in connection with the processing of confidential patient information. This committee might well explore with stakeholders the experiential dimensions of data use with a view to informing the advice that it will give.

Beyond providing a clear guide through the experience, researchers and regulators could work more proactively to support citizens in recognising the collective and potentially transformative experience of *becoming* a research participant. This is with the caveat that it is not on the basis of engendering such experience into each and every citizen, while recognising that experience is at once individual and collective. A common feature of accounts of liminality is that it is a *shared* experience. Normatively, the potential power of liminality arises from a common human happening that is a great leveller—it puts us all in the same boat. We can recognise value in ourselves, and in others, and in the status change that is being brought about. If that change is to contribute to a collective, common good, then liminality has a tentative role to play. Opportunities could be provided of access to positive models of involvement in health research, as well as occasions to share and learn from the experiences of other research participants,^121^ researchers, and regulators.

Finally, what of the relationship between law and liminality in all of this? The preceding analysis would suggest that this is an uneasy union. When spontaneous liminality is triggered by crisis—however defined—then law is often called upon to fill the void. There is a plethora of examples of this happening in the medico-legal sphere, and the reaction is understandable because law can represent certainty, structure, and directed agency, while spontaneous liminality is typified by uncertainty, anti-structure, and an absence of agency (or even a perceived failure of agency). If we seek to cast law as the Representative of Order, we must equally be alert to it assuming the guise of Trickster, or at least that it is perceived as such (as the example of *care.data* likely illustrates). However, even when the motive behind legal intervention is well intentioned, we should not lose sight of the impositional character of law, which, by its nature, seeks to establish structures, fix regulatory objects, assign responsibilities, and attribute liabilities.^122^ A regulatory space that is constructed and defined solely in such terms misses something very crucial in-between. This analysis suggests that it is possible to envision in-between spaces where people experience unsettling—but also potentially empowering—changes of status and influence. Such uncontrolled spaces—in the sense of spaces that support and facilitate genuinely open-ended engagement and interaction—can be affirming and productive, and could take regulation in new and more effective directions. The real question for law is whether it can envision and support the creation of genuinely liminal regulatory spaces that go against its own inherent tendencies, and yet which, as has been demonstrated herein, would capture something very human about the conduct of human health research.

## VI. CONCLUSION

This article has examined what it means to miss the spaces in-between the current regulatory spaces created in the health research environment. In contradistinction to law’s tendency to create legal silos of regulatory attention around artificial and fixed ‘objects’, the argument has been made that we must reimagine regulatory space and time as an holistic enterprise which places human experience at the centre and which focuses equally on the human *subject*. The concept of liminality allows us to do so, and it reveals a plethora of insights about the workings and challenges of current health research regimes. The ironic insight is that current structures, themselves, create the liminal experiences discussed above. However, if we fail to see involvement in health research as an essentially transformative experience, then we blind ourselves to many of the human dimensions of health research. More worryingly, we run the risk of overlooking deeper explanations about why some projects fail and why the entire enterprise continues to operate sub-optimally. While this is not to suggest that liminality provides complete solutions, it does invite radical rethinking about *how* health research is experienced. This, in turn, demands that law and legal architectures accommodate liminal regulatory spaces, even when these appear antithetical to the commonly accepted social roles of law itself.

## Footnotes

1 See, in particular, R Baldwin, M Cave and M Lodge, *Understanding Regulation: Theory, Strategy, and Practice* (2nd edn, OUP 2012) and F Vibert, *The New Regulatory Space: Reframing Democratic Governance* (Edward Elgar 2014).

2 Substantially amended and extended in 2008 through the Human Fertilisation and Embryology Act 2008.

3 See GT Laurie and SHE Harmon, ‘Through the Thicket and Across the Divide: Successfully Navigating the Regulatory Landscape in Life Sciences Research’ in E Cloatre and M Pickersgill (eds), *Knowledge, Technology and Law* (Routledge 2015) 121.

4 Council Regulation (EU) 536/2014 of 16 April 2014 on clinical trials on medicinal products for human use, and repealing Directive 2001/20/EC [2014] OJ L158/1.

5 Council Directive 95/46/EC of 24 October 1995 on the protection of individuals with regard to the processing of personal data and on the free movement of such data [1995] OJ L281/31. The General Data Protection Regulation was adopted by the Council of the EU on 8 April 2016 and by the European Parliament on 14 April 2016. Content and legislative history here: < http://ec.europa.eu/justice/data-protection/reform/index_en.htm> accessed 30 April 2016.

6 Council Directive 90/385/EEC of 20 June 1990 on the approximation of the laws of the Member States relating to active implantable medical devices [1990] OJ L189/17; Council Directive of 93/42/EEC concerning medical devices [1993] OJ L169/1; and Council Directive of 27 October 1998 on in vitro diagnostic medical devices [1998] OJ L331/1. Proposals for reform can be followed here: <http://ec.europa.eu/growth/sectors/medical-devices/index_en.htm> accessed 30 April 2016.

7 Council Regulation 1394/2007 of 13 November 2007 on advanced therapy medicinal products and amending Directive 2001/83/EC and Regulation (EC) No 726/2004 [2007] OJ L324/121. Proposals for reform can be followed here: <http://ec.europa.eu/health/human-use/advanced-therapies/index_en.htm> accessed 30 April 2016.

8 For a discussion of top-down governmental approaches and a range of alternatives, see C Lyall, T Papaioannou and J Smith (eds), *The Limits to Governance: The Challenge of Policy-Making for the New Life Sciences* (Ashgate 2009).

9 See, eg the Council of Europe, Convention on Human Rights and Biomedicine: Oviedo Convention (4 April 1997), and the increasingly legalistic World Medical Association, Declaration of Helsinki: Ethical Principles for Medical Research Involving Human Subjects (10th edn, 2013).

10 Classically, see O O’Neill, *Autonomy and Trust in Bioethics* (CUP 2002). More recently, see, G Laurie and others, *A Review of Evidence Relating to Harm Resulting from Uses of Health and Biomedical Data* (Nuffield Council on Bioethics 2015), available at: <http://nuffieldbioethics.org/project/biological-health-data/evidence-gathering/> accessed 30 April 2016.

11 s 111(1).

12 s 111(2).

13 This is true both of hard law features, such as those discussed in the text, and soft laws such as the myriad guidance documents from professional bodies such as the British Medical Association and General Medical Council, as well as practice statements or Standard Operating Procedures from regulators, such as the Health Research Authority. A more recent phenomenon has witnessed increased interest from funders, such as the Wellcome Trust and the Economic and Social Research Council, through the issuing of guidance on what they expect of researchers in ethical terms.

14 This is not to be confused with the concept of ‘boundary objects’, see S Leigh Star, ‘This is Not a Boundary Object: Reflections on the Origin of a Concept’ (2010) 35 Sci, Technol & Human Values 601.

15 The term ‘ecosystem’ is used here as a shorthand to encompass the complex network of actors and evolutionary dynamics that typify and drive forward a particular sector of activity. It is a term that is deployed in health research policy circles; see, for example, JN Thompson and J Moskowitz, ‘Preventing the Extinction of the Clinical Research Ecosystem’ (1997) 278(3) J Am Med Ass 241, and M Swan, ‘Crowdsourced Health Research Studies: An Important Emerging Complement to Clinical Trials in the Public Health Research Ecosystem’ (2012) 14(2) J Med Internet Res Mar-Apr: e46.

16 See also, LA Bygrave, ‘Information Concepts in Law: Generic Dreams and Definitional Daylight’ (2015) 35(1) OJLS 91.

17 This is not to deny the considerable efforts of regulators to devise memoranda of cooperation to work better together, for example Memorandum of Understanding (MoU) between the Human Tissue Authority and the Human Fertilisation and Embryology Authority (2013), available at: < http://www.hfea.gov.uk/docs/HFEA_HTA_MoU_and_JWP.pdf> accessed 30 April 2016.

18 T Caulfield and others, ‘The Stem Cell Research Environment: A Patchwork of Patchworks’ (2009) 5(2) Stem Cell Rev 82.

19 Compare the US National Research Act 1974, in which ‘research subjects’ are legal constructs.

20 For discussion of a possible ontological trend in other literatures, see S Woolgar, ‘The Wrong Bin Bag: A Turn to Ontology in Science and Technology Studies?’ (2013) 43(3) Social Studies of Science 321.

21 G Laurie and E Postan, ‘Rhetoric or Reality: What is the Legal Status of the Consent Form in Health-related Research?’ (2013) 21(3) Med L R 371.

22 For example, DC English, ‘Valid Informed Consent: A Process, Not a Signature’ (2002) Am Surgeon 45, and P Allmark and S Mason, ‘Improving the Quality of Consent to Randomised Controlled Trials by Using Continuous Consent and Clinician Training in the Consent Process’ (2006) 32 J Med Ethics 439.

23 B Thomassen, *Liminality and The Modern: Living Through the In-Between* (Ashgate 2014), 220, citing A Horvarth, *Modernism and Charisma* (Palgrave MacMillan 2013) 195, and H Wydra, ‘The Liminal Origins of Democracy’ (2009) 2(1) International Political Anthropology 91.

24 R Girard, *Violence and the Sacred* (Johns Hopkins University Press 1979) provided a sweeping account of mimetic desire in ancient and more recent cultures.

25 Thomassen (n 23) 220.

26 ibid 221.

27 P Ohm, ‘Broken Promises of Privacy: Responding to the Surprising Failure of Anonymization’ (2010) 57 UCLA Law Rev 1701, and more recently Nuffield Council on Bioethics, The Collection, Linking and Use of Data in Biomedical Research and Health Care: Ethical Issues (Nuffield Council on Bioethics: London, 2015), paras 4.15–4.29.

28 W Lowrance, *Privacy, Confidentiality and Health Research* (CUP 2012), and R Al-Shahi and C Warlow, ‘Using Patient-Identifiable Data for Observational Research and Audit’ (2000) 321 BMJ 1031.

29 The fact that this might not be important to some people, subjectively, is not the point at issue.

30 NHS England and others, Frequently Asked Questions: *care.data* Guide for GP Practices, (2013).

31 To be specific, data could be identifiable while used in the HSCIC but it would not be disseminated in identifiable form.

32 NHS Act 2006, s 251.

33 ibid.

34 P Carter, GT Laurie and M Dixon-Woods, ‘The Social Licence for Research: Why care.data Ran into Trouble’ (2015) 41 J Med Ethics 404.

35 A van Gennep, *The Rites of Passage* (University of Chicago Press 1960) 21. Emphasis as in original text.

36 ibid 189: ‘Forms [of rites of passage] often vary, but the function is similar.’

37 ibid x.

38 ibid 13.

39 A Szakolczai, ‘Liminality and Experience: Structuring Transitory Situations and Transformative Events’ in A Hovarth, B Thomassen and H Wydra (eds), *Breaking Boundaries: Varieties of Liminality* (Berghahn Books 2015) 18.

40 P Stenner and E Moreno-Gabriel, ‘Liminality and Affectivity: The Case of Deceased Organ Donation’ (2013) 6 Subjectivity 229.

41 ibid 241.

42 It is crucial to recognise the work of critical anthropologists in the post-colonial and capitalist industrial era who have highlighted the importance in ethnographic writing ‘ … [to] struggle self-consciously to avoid portraying abstract … a-historical “others”’, see J Clifford, ‘On Ethnographic Authority’ (1983) 2(1) Representations 118, 119. See also, J Clifford and GE Marcus, *Writing Culture: The Poetics and Politics of Ethnography* (University of California Press 1986).

43 See, generally, the works discussed in sub-Section III.B below.

44 V Turner, *The Ritual Process: Structure and Anti-Structure* (Aldine Transaction 1969). See also, beyond the tribal studies context, V Turner, *From Ritual to Theatre: The Human Seriousness of Play* (PAJ Books 1982), V Turner, *The Anthropology of Performance* (PAJ Books 1986), BC Alexander, *Victor Turner Revisited: Ritual as Social Change* (1991) and G St. John, *Victor Turner and Contemporary Cultural Performance* (Berghahn Books 2008).

45 Turner, *The Ritual Process,* ibid 95.

46 ibid.

47 See generally, all materials referenced in n 23.

48 Turner, *The Ritual Process*, (n 44) 116.

49 A Szakolczai, ‘Liminality and Experience: Structuring Transitory Situations and Transformative Events’ (2009) 2(1) International Political Anthropology 141, 147.

50 I borrow here from the work of Peter Fitzpatrick on ‘law as a matter of myth’ in P Fitzpatrick, *The Mythology of Modern Law* (Routledge 1992). I am grateful to one of the anonymous referees for bringing this work to my attention.

51 [1986] AC 112 (HL).

52 See *Re R (a minor)(wardship: medical treatment)* [1992] Fam 11 and *Re W (a minor)(medical treatment)* [1992] 4 All ER 627.

53
*Re T (adult)(refusal of medical treatment)* [1992] 4 All. ER 649, together with *Re MB (an adult: medical treatment)* [1997] 8 Med LR 217.

54 See for example, S Elliston, *The Best Interests of the Child in Healthcare* (Routledge 2007).

55 Compare J Tripp and R Viner, ‘Sexual Health, Contraception and Teenage Pregnancy’ (2005) 330 BMJ 590, and R Taylor, ‘Reversing the Retreat from *Gillick*? *R (Axon) v Secretary of State for Health*’ (2007) 19 CFLQ 81.

56 Building on the point noted in n 29, it is important not to suggest that the subjective experience of the subject is necessarily transformative *for them*.

57 K Martin-McDonald and D Biernoff, ‘Initiation into a Dialysis-dependent Life: An Examination of Rites of Passage’ (2002) 29(4) Nephrology Nursing Journal 347.

58 M Little and others, ‘Liminality: A Major Category of the Experience of Cancer Illness’ (1998) 47(10) Social Science and Medicine 1485, and K Thompson, ‘Liminality as a Descriptor for the Cancer Experience’ (2007) 15(4) Illness, Crisis and Loss 333.

59 E Blows and others, ‘Liminality as a Framework for Understanding the Experience of Cancer Survivorship: A Literature Review’ (2012) 68(10) J Advanced Nursing 2155.

60 H Braude, ‘Normativity Unbound: Liminality in Palliative Care Ethics’ (2012) 33 Theor Med Bioeth 107.

61 K Froggatt, ‘Rites of Passage and the Hospice Culture’ (1997) 2(2) Mortality 123, 127.

62 Thomassen (n 23), carves out both a narrow and wide conceptualisation of liminality. The narrow is the sense of the middle stage of life’s passages as identified by van Gennep, while the wider notion ‘ … captures something essential about the imprecise and unsettled situation of transitoriness’. Here he cites Horvarth (n 23) 10.

63 As he states, (n 23) 38: ‘Liminal spaces and moments are key to personal and social development, anywhere in the world. It is via the liminal that persons and groups are taken apart, recomposed and regenerated.’ Moreover, he continues, sagely: ‘van Gennep was right.’

64 ibid 94–99.

65 World Health Organization Ebola Response Team, ‘Ebola Virus Disease in West Africa—The First 9 Months of the Epidemic and Forward Projections’ (2014) 371 N Engl J Med 1481, and G Laurie and KG Hunter, ‘Mapping, Assessing and Improving Legal Preparedness for Pandemic Flu in the United Kingdom' (2009) 10 Med Law Int 101.

66 Thomassen (n 23) 99.

67 Szakolczai (n 49).

68 See, in particular, Girard (n 24).

69 See L Ellis, ‘Trickster: Shaman of the Liminal’ (1993) 5(4) Studies in American Indian Literatures 55. Also, discussion in Thomassen (n 23) 103–05.

70 Turner, n 44, (1982), 104.

71 Turner, *The Ritual Process* (n 44) and text.

72 ibid 97. He prefers communitas to community because the latter implies ‘area of common living’ and this is not necessary for communitas.

73 E Turner, *Communitas: The Anthropology of Collective Joy* (Palgrave Macmillan 2012) suggests that communitas should be distinguished from Durkheim’s solidarity: ‘ … which is a bond between individuals who are collectively in opposition to some other group … But in the way communitas unfolds, people’s sense is that it is for everybody- humanity, bar none. In communitas moments in the heart of revolutions it is the same … ’, 5.

74 Turner, *The Ritual Process* (n 44) 97.

75 P Stoller, *Stranger in the Village of the Sick: A Memoir of Cancer, Sorcery, and Healing* (Beacon Press 2004) 184.

76 Again, this is not to suggest that this is naturally or necessarily the subjective experience. Other research, currently being conducted on the Liminal Spaces Project with rare diseases patient groups, suggests that on some occasions this kind of mobilisation can amplify disease burden: people have to deal with their own disease *and* that of others. Thanks to Samuel Taylor-Alexander for this point.

77 Turner (n 73) xii.

78 ibid, 4.

79 See generally, N Doorn and others, ‘Early Engagement and New Technologies’ (2013) 16 Philos Eng and Technol, Special Issue. See too, S Hogarth, ‘Regulatory Experiments and Transnational Networks: The Governance of Pharmacogenomics in Europe and the United States’ (2012) 25(4) Innovation: The Eur J Social Sci Res 441.

80 This is a typical definition taken from the Engineering and Physical Sciences Research Council (EPSRC) website: <https://www.epsrc.ac.uk/funding/howtoapply/routes/network/ideas/whatisasandpit/> accessed 30 April 2016.

81 Turner, n 44, in his later works explored the importance and potential value of ’play’ in opening up the creative potential of liminal spaces.

82 This is not the same as saying that it is *unstructured*.

83 This function could also, partially, be addressed through policy and guidance.

84 n 4, ch V, arts 28–35.

85 See, in the UK context, Health Research Authority, Standard Operating Procedures for Research Ethics Committees (v 6.1 2015). The term ‘consent’ appears no fewer than 209 times.

86 M Wilson, ‘Nyahyusa Ritual and Symbolism’ (1954) 56(2) Am Anthropol 228, 241.

87 See Laurie and Postan (n 21) and associated texts.

88 n 22.

89 See JJ Ladge, JA Clair and D Greenberg, ‘Cross-Domain Identity Transition during Liminal Periods: Constructing Multiple Selves as Professional and Mother during Pregnancy’ (2012) 55(6) Academy of Management J 1449.

90 See also, A Mol, *The Logic of Care: Health and the Problem of Patient Choice* (Routledge, 2008).

91 Ladge and others (n 89).

92 See, eg the Centre for Longitudinal Studies, Home of the 1958, 1970, Millennium and Next Steps Cohort Studies: <http://www.cls.ioe.ac.uk/> accessed 30 April 2016.

93 See Avon Longitudinal Study of Parents and Children: <http://www.bristol.ac.uk/alspac/researchers/> accessed 30 April 2016.

94 Personal correspondence, HRA Applications Manager (2015).

95 The Scottish Health Informatics Programme, sponsored by the Wellcome Trust, ran from 2009–2013 and was tasked with improving data linkage for research in Scotland: see, <http://www.scot-ship.ac.uk/sites/default/files/Reports/SHIP_BLUEPRINT_DOCUMENT_final_100712.pdf> accessed 30 April 2016.

96 The work of SHIP has now been absorbed into the UK-wide Farr Institute: <http://www.farrinstitute.org/> accessed 30 April 2016.

97 I discuss this further with colleagues A Ganguli-Mitra, E Dove and S Taylor-Alexander in ‘Reconfiguring Social Value in Health Research Through the Lens of Liminality’, forthcoming.

98 N Stephens, P Atkinson and P Glasner, ‘Documenting the Doable and Doing the Documented: Bridging Strategies at the UK Stem Cell Bank’ (2011) 41(6) Social Studies of Science 791, 807.

99 See, eg the statutory obligation in New Zealand for government departments to exercise regulatory stewardship of the legislation administered by the department, where ‘stewardship’ is defined as: ‘ … active planning and management of medium- and long-term interests, along with associated advice’: s 32 of the State Sector Act 1988.

100 I am grateful to the insights of Paul Stenner for this expression used at a workshop of our Liminal Spaces project, held in Edinburgh in May 2015, details here: <http://www.liminalspaces.ed.ac.uk/2015/07/01/190/> accessed 29 July 2016. The event was held as part of the Wellcome Trust funded project ‘Confronting the Liminal Spaces of Health Research Regulation’, Award No: WT103360MA.

101 I use this term in contradistinction to the notion of ‘boundary objects’ developed by Star (n 14).

102 S Merrill Squier, *Liminal Lives: Imagining the Human at the Frontiers of Biomedicine* (Duke University Press 2004) 4.

103 D Hall, ‘Reflecting on Redfern: What Can We Learn from the Alder Hey Story?’ (2001) 84 Arch Dis Child 455.

104 For criticisms, see Academy of Medical Sciences, Personal Data for Public Good: Using Health Information in Medical Research (2011); Academy of Medical Sciences, A New Pathway for the Regulation and Governance of Health Research (2012); R Thomas and M Walport, ‘Data Sharing Review Report’ (2008).

105 This concept is discussed further by Horvarth (n 23) and Szakolczai (n 49).

106 See Ohm (n 27).

107 Lowrance (n 28).

108 cf J Walle, ‘The Legal Status of Body Parts: A Framework’ (2011) 31(4) OJLS 783.

109 The Independent, ‘Blood Donors in Sweden Get a Text Message Whenever Their Blood Saves Someone's Life’, 10 June 2015, available at < http://www.independent.co.uk/news/world/europe/blood-donors-in-sweden-get-a-text-message-whenever-someone-is-helped-with-their-blood-10310101.html> accessed 30 April 2016.

110 The term is used deliberately here in contradistinction to volunteer research ‘participants’.

111 n 89, above.

112 The paradigmatic example of the Trickster is the savior dictator who emerges from the context of a country in crisis to save the day. Consider the experiences of Germany in the post-World War One era and the rise of the Chancellor who took over power in 1933. It is often in the Trickster’s interests that a chaotic, liminal state continues.

113 The Independent Information Governance Oversight Panel’s Report to the *care.data* Programme Board on the *care.data* Pathfinder Stage (2014), available at: <https://www.gov.uk/government/uploads/system/uploads/attachment_data/file/389219/IIGOP_care.data.pdf> accessed 30 April 2016.

114 The list was: (i) Use of the data and clear explanation of purposes; (ii) A secure data facility to allow approved applicants to use and analyse data; (iii) Transparency—HSCIC to update and maintain its register of data releases; (iv) Confidentiality—The Code of Practice and fair processing information to be provided on the HSCIC website; (v) Control of data—NHS England and HSCIC to be joint Data Controllers; (vi) Business case development—to be developed and endorsed by the Programme Board before data extraction; (vii) Evaluation—a full evaluation at the end of the Pathfinder stage will inform the wider rollout of *care.data*. At the time of submission the rollout had not yet occurred (May 2016).

115 National Data Guardian for Health and Care, *Review of Data Security, Consent and Opt-Outs*, 6 July 2016, available here: <https://www.gov.uk/government/uploads/system/uploads/attachment_data/file/535024/data-security-review.PDF> accessed 6 July 2016. Also, Care Quality Commission, Safe Data, Safe Care, 6 July 2016, available here: <http://www.cqc.org.uk/sites/default/files/20160701%20Data%20security%20review%20FINAL%20for%20web.pdf> accessed 6 July 2016.

116 See Written Statement to Parliament, Review of Health and Care Data Security and Consent, 6 July 2016, available at: <https://www.gov.uk/government/speeches/review-of-health-and-care-data-security-and-consent> accessed 6 July 2016.

117 Citing Turner, *The Ritual Process* (n 44).

118 Thomassen (n 23) 92.

119 n 94, and text.

120 Further gratitude is due to Stenner for this distinction, (n 100). An additional liminal category might be entities that are between established ontological well-regulated categories. For example, consider biobricks which neither emerge from crisis nor are they in need of guidance. These are DNA sequences designed to comply with multiple assembly engineering standards in a effort to build higher-order biological systems: see further the Biobricks Foundation, available at < http://biobricks.org/> accessed 30 April 2016.

121 See Ladge and others (n 89) 1467.

122 Poignantly, consider the words of Szakolczai (n 39) 31: ‘Institutions, laws, and structures will always be deficient, failing us just when we need the most protection and security.’

